# Self-Reported Late Effects in Childhood Cancer Survivors in Kenya

**DOI:** 10.4314/ahs.v24i3.27

**Published:** 2024-09

**Authors:** Jesse Lemmen, Susan Mageto, Festus Njuguna, Nancy Midiwo, Sandra Langat, Terry Vik, Gertjan Kaspers, Saskia Mostert

**Affiliations:** 1 Pediatric Oncology, Emma Children's Hospital, Amsterdam UMC, Vrije Universiteit Amsterdam, Amsterdam, the Netherlands; 2 Princess Máxima Center for Pediatric Oncology, Utrecht, the Netherlands; 3 Academic Model Providing Access to Healthcare (AMPATH), Eldoret, Kenya; 4 Pharmacology, School of Health Sciences, Kisii University, Kisii, Kenya; 5 Department of Child Health and Pediatrics, Moi University, Eldoret, Kenya; 6 Pediatrics, Indiana University School of Medicine, Indianapolis, Indiana, USA; Academic Model Providing Access to Healthcare (AMPATH), Eldoret, Kenya

**Keywords:** Pediatrics, survivorship, neoplasms, aftercare

## Abstract

**Background:**

The number of children surviving cancer in low and middle-income countries is expected to grow in the coming years. Knowledge about late effects and follow-up preferences in Kenya is lacking.

**Objectives:**

This study assessed self-reported late effects in Kenyan childhood cancer survivors and explored their preferences for survivorship care.

**Methods:**

Childhood cancer survivors, having successfully completed treatment for at least one year, were interviewed using semi-structured questionnaires during clinic or home visits between 2021-2022. Medical records were reviewed for patient and treatment characteristics.

**Results:**

Twenty-six survivors of hematological malignancies (n=19, 73%), solid tumors (n=6, 23%), unknown tumor type (n=1, 4%), were interviewed. Most survivors (n=19, 73%) solely received chemotherapy and one survivor (4%) was irradiated. Median time since treatment completion was seven years. Fifteen survivors (58%) were previously lost to follow-up. Many survivors (n=19; 73%) self-reported late effects, predominantly pain and fatigue. Survivors (n=11, 42%) were limited in daily life activities: physical work (n=10, 38%), personal care (n=6, 23%), social activities (n=6, 23%). Eight survivors (31%) recalled being informed about late effects. Some survivors experienced a negative attitude toward cancer in regional hospitals. Follow-up duration was longer among informed patients (p=0.043). Survivors recommended education and survivor meetings and preferred their follow-up to be done at the referral center.

**Conclusions:**

Kenyan childhood cancer survivors self-report late effects, comparable in frequency, nature and severity to other survivors worldwide. Survivors and healthcare providers require education about the lifelong impact of childhood cancer and should have access to survivorship expertise to continue follow-up.

## Introduction

In high-income countries (HIC), childhood cancer survival rates have increased to 80% and most children with cancer will become adults.^1^,^2^ Less than 35% of children with cancer in low and middle-income countries (LMIC) are currently cured.^3^,^4^ However, successfully treating common and curable pediatric malignancies in LMIC has become a more realistic goal through the implementation of cost-effective strategies that tackle the many challenges surrounding cancer care.^5^-^8^ As a result, the number of childhood cancer survivors in low-and-middle-income (LMIC) is also expected to grow in the coming years.

Cancer treatment is associated with acute but transient side effects such as neutropenic fever or nausea.^9^ Survivorship care, by contrast, involves the management of potentially chronic adverse events following cancer treatment, ranging from subsequent malignancy to cardiac, endocrine, and neurological disorders.^10^-^12^ Surveillance of these late treatment effects generally starts within five years after treatment completion. Morbidity can appear up to decades after the initial cancer diagnosis.^11^,^13^ To meet the needs of both patients and healthcare givers, multiple national and international guidelines have emerged for follow-up of late effects.^13^,^14^ Risk profiles help to distinguish patients who require close monitoring, for example children exposed to high-dose cytostatic agents, from those who do not. The International Guideline Harmonization Group has combined the plethora of existing guidelines into recommendations that apply globally.^15^ However, the core group of this initiative consists only of members from high-income countries, and their consensus standard of care may not be implementable in LMIC.

Knowledge about long-term follow-up and survivorship care in LMIC such as Kenya is lacking. Most reports have focused on a single diagnosis or late effect, including patients who were followed for a limited time post-treatment.^16^-^18^ Understanding late therapy effects of patients with different ethnic, genetic, and environmental backgrounds may guide the development of adapted treatment and monitoring plans.^19^

This study aimed to assess the prevalence of self-reported late effects in childhood cancer survivors and explore their preferences for follow-up care. The challenges that young adult cancer survivors in Western Kenya encounter after cancer treatment completion will provide helpful information to guide current and future survivors and establish the necessary long-term care.

## Methods

### Setting

Kenya is an East African lower middle-income country with a gross domestic product per capita of 2099 United States Dollars (USD). Thirty-six percent of the population lives below the international poverty line. Kenya has an estimated population of 52 million, including 20 million children under 14 years.^20^,^21^ English and Kiswahili are the two official languages.^22^

The study was conducted at Moi Teaching and Referral Hospital (MTRH), a referral and training hospital offering comprehensive pediatric care. MTRH serves primarily Western Kenya, accounting for half of the country's population.^23^ More than 200 children are diagnosed with malignancies at MTRH yearly. Two pediatric oncologists supervise the department; treatment options include chemotherapy, (neuro)surgery, and, since 2021, radiotherapy.^24^ Previously, patients who required radiotherapy were sometimes referred to other facilities. The National Health Insurance Fund (NHIF) covers health expenditures for voluntary subscribers or those formally employed by deducting a monthly charge. Depending on income, the monthly fee amounts to approximately four USD.^25^ Enrollment provides access to a (limited) package, including the essential services to treat childhood cancer.^26^,^27^ A revision of the NHIF, the Social Health Insurance Fund (SHIF), is expected to be implemented in 2024.^28^

### Study Design

This exploratory, descriptive study was performed using a semi-structured questionnaire. Participants were childhood cancer survivors (≥18 years), diagnosed between January 1st 2010 and December 31^st^ 2019, who had completed cancer treatment and had at least one year of event-free survival post-treatment completion. Event-free survival was defined as the absence of any treatment failure (abandonment of treatment, progressive or relapsed disease, death).

Participants were interviewed in one session by a researcher capable of performing the interview in English or Kiswahili. The estimated length of time per interview was 60-90 minutes. Interviews occurred at the hospital or home between November 2021 and October 2022. Informed consent was obtained.

The researcher-designed questionnaire contained structured questions and several open questions. The questionnaire's items were derived from an extensive literature study and expert opinions of a panel of Kenyan, North American, and Dutch doctors.^29^-^31^ The questionnaire was available in English and Kiswahili. First, the English version of the questionnaire was translated into Kiswahili. Second, the Kiswahili version of the questionnaire was translated back to English, and a comparison between the versions was made to detect any inaccuracies. The questionnaire was pilot-tested on five childhood cancer survivors. After evaluating this pilot, a few questions were redefined and clarified, and additional questions were added.

Eighteen categories of self-reported late effects were distinguished: pain, fatigue, ophthalmologic problems, gastrointestinal problems, dental problems, orthopedic problems, shorter stature than siblings, neurological problems, psychological problems, cognitive problems, renal problems, ear/nose/throat problems, cardiac problems, respiratory dysfunction, endocrine dysfunction, subsequent malignancy, and other problems. The respondent categorized the severity of self-reported late effects according to subjective experience as mild, moderate or severe.. Notably, the self-reported prevalence and severity of late effects or symptoms were thus not verified by physical examinations or additional investigations.

Socio-demographic, patient, and treatment characteristics were retrieved from the medical records of the childhood cancer survivors: sex, date of birth, diagnosis, date of diagnosis, type of cancer (hematological/solid tumors), date of starting treatment, date of treatment completion, date of last follow-up, type of treatment (chemotherapy/surgery/radiotherapy), health-insurance status, follow-up duration after completion of treatment.

By definition, information status refers to recalling being informed about the late effects of cancer treatment at MTRH before completion of treatment. Performance status refers to restrictions of personal care, physical work, social activities, daily chores, and school (performance and attendance) in the last four weeks. Follow-up duration reflected the time between completion of treatment and the last hospital visit. A survivor was considered “in follow-up” if the last hospital visit occurred less than two years before the interview. A survivor was “lost to follow-up” if the last hospital visit was two years or more before the interview.

A summary of the response to the open questions was noted on the interview form directly during the interview. No audio recordings were made.

Anonymity and confidentiality were guaranteed. The study protocol was approved by the Institutional Research and Ethics Committee (FAN: 0004007).

### Data Analysis

Data were transferred from the paper questionnaire form into a digital data storage (Castor EDC). Data were extracted and analyzed in IBM SPSS Statistics 26. Frequencies and descriptive statistics were calculated. Qualitative data derived from the open question about recommendations for guidance and follow-up of survivors were color-coded per statement in Microsoft Office Word 2016. Codes were grouped, and underlying themes identified. Differences in follow-up duration or experience of self-reported late effects and various socio-demographic and clinical patient and treatment characteristics (sex, age at diagnosis, type of cancer, treatment type, travel time, information status, health-insurance status, age at interview, performance status, time since completion of treatment, follow-up duration, interview location) were compared using chisquared test, Fisher's exact test and independent t-test. Statistical significance was set at a two-sided P value of less than 0.05.

## Results

### Survivor Characteristics

From January 2010 until December 2019, 1472 children were newly diagnosed with a malignancy at MTRH. Based on survival rates reported in a previous study, approximately 450 of these children successfully finished treatment.^32^ Of these children, an estimated 100 were ≥18 years old at the time of the interview and had at least one year of event-free survival after completion of treatment.

**Figure 1:**
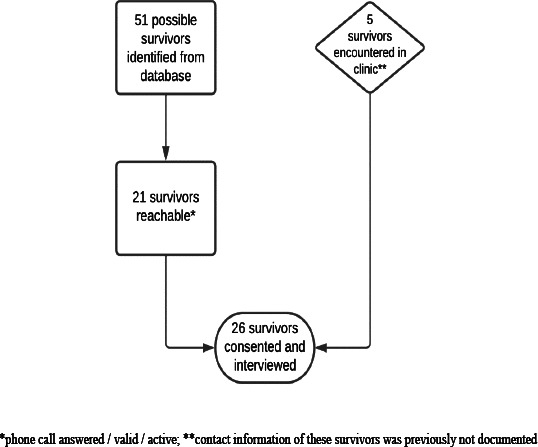
Participant selection flow diagram

Contact details were available for 51 eligible childhood cancer survivors (Figure 1). The phone number of four survivors was not documented. Twenty-one of these survivors could be reached by phone. Phone numbers of the remaining 30 survivors were either no longer valid/active, or the survivors did not answer their phones. In addition, five survivors were recruited who attended MTRH for follow-up clinic visits. In total, 26 survivors were invited to participate in this study. All consented to participate upon receiving study information. The survivors were interviewed either at the hospital (54%) or during a home visit (46%).

More males (62%) were interviewed (Table 1). Median age at interview was 20 years (IQR 18.0 – 22.3) and 12.5 years (IQR 10.0 – 14.3) at diagnosis. Distinguished types of cancer were hematological malignancies (73%), solid tumors (23%), and unknown (4%). The most prevalent diagnoses were non-Hodgkin lymphoma (31%), Hodgkin lymphoma (23%), and acute lymphoblastic leukemia (19%).

**Table 1:** Socio-demographic and clinical characteristics of childhood cancer survivors (n=26)

Characteristics	N / Median (% / IQR)
**Sex**	
Male	16 (62%)
Female	10 (39%)

**Age at interview**	20.0 (18.0 – 22.3)
**Age at diagnosis**	12.5 (10.8 – 14.3)

**Diagnosis**	
Non-Hodgkin lymphoma	8 (31%)
Hodgkin lymphoma	6 (23%)
Acute lymphoblastic leukemia	5 (19%)
Osteosarcoma	3 (12%)
Germ cell tumor	2 (8%)
Kaposi sarcoma	1 (4%)
Unknown	1 (4%)

**Treatment**	
Chemotherapy	19 (73%)
Chemotherapy + surgery	5 (19%)
Chemotherapy + surgery + radiotherapy	1 (4%)
Unknown	1 (4%)

**Follow-up duration**	
<1 years	7 (27%)
1 – 3 years	5 (19%)
3 – 5 years	6 (23%)
>5 years	8 (31%)

**Follow-up status**	
Lost to follow-up	15 (58%)
In follow-up	11 (42%)

**Health-insurance during treatment**	
Prior to diagnosis	11 (42%)
Activated during treatment	6 (23%)
Unknown	9 (35%)

**Health-insurance after treatment**	
Yes	18 (69%)
No	8 (31%)

### Childhood Cancer Treatment

Most survivors (n=19, 73%) had solely received chemotherapy and one survivor (4%) was irradiated. Chemotherapy regimens contained alkylating agents (60%) and anthracyclines (80%) in combination with other agents. Follow-Up Most survivors (92%) were initially followed up after completion of treatment. Median time since completion of treatment was 7.2 years (IQR 5.1 – 9.9). Median duration of follow-up was 3.3 years (IQR 0.7 – 5.8), with eight survivors (31%) in follow-up longer than five years. Median follow-up time was equally distributed between men (3.1 years) and women (3.3 years). Only 11 survivors had attended the follow-up clinic within the last two years before the interview. Fifteen survivors (58%) were previously lost to follow-up. Follow-up time was significantly longer for patients who did versus did not recall being informed about late effects (mean 5.6 years vs 2.9 years; p=0.043 [CI 95 0.10 – 5.28]). Patients diagnosed when more than 12 years had a significantly shorter follow-up time than those diagnosed when less than 12 years of age (mean 2.3 years vs 5.2 years; p=0.015 [CI 95 0.64 – 5.30]).

### Health Insurance Status

Forty-two percent of survivors were enrolled in health insurance prior to treatment, and for 23%, insurance was activated during treatment. At the time of the interview, 69% of survivors were health insurance subscribers, and 31% were not. Most of these non-subscribers (88%) never registered for health insurance when they became of adult age, and one survivor had no finances to subscribe.

### Transportation to MTRH

Survivors lived <50 km (15%), 50-100 km (31%) and >100km (54%) away from MTRH. Travel time to MTRH was less than one hour (12%), one to three hours (54%) or more than three hours (35%). They used the following modes of transport to the hospital: public transport (89%), private car (8%), renting a vehicle (4%), walking (4%). Survivors felt that travelling was time-consuming (65%), expensive (65%), and difficult (50%).

### Self-reported Late Effects

In total, 19 survivors (73%) self-reported late effects (Table 2). The most prevalent self-reported late effects were pain (42%), fatigue (35%), ophthalmologic problems (31%), gastrointestinal problems (27%), orthopedic problems (27%), and dental problems (27%). Pain was recurrent in 82% and longstanding in 18%. No secondary malignancies were reported. Seventy-one health conditions were identified, of which 27% were mild, 50% moderate, and 23% severe. Most severe symptoms were observed in pain, gastrointestinal, orthopedic, and dental categories. Late effects were reported in all assessed categories except for endocrine conditions. Nineteen percent had a self-reported short stature in comparison with their siblings. The number of self-reported late effects per survivor varied from zero to eight (median 2.5, IQR 0-4). The maximum grade of self-reported late effects per survivor was mild (6%), moderate (39%), and severe (56%). Nineteen percent of survivors had ever consulted a psychologist or a psychological counselor. Among survivors with psychological or cognitive problems, this was 33% and 50%, respectively. Patients with a limited performance status had a significantly greater number of self-reported late effects (mean difference 3.2; p<0.001 [CI 95 1.69 – 4.65]).

**Table 2:** Overview of self-reported late effects in childhood cancer survivors (n=26)

Health conditions	N (%)	Severity		Symptoms
**Pain**	11 (42%)	Mild	2	Jaw pain
		Moderate	6	Back pain
		Severe	3	Headache; pain shoulder/legs; pain at amputated leg

**Fatigue**	9 (35%)	Mild	3	Tired at work; cannot run/walk like
		Moderate	6	peers
		Severe	0	Exhausted after intensive activities; due to heart problems; more energy to move

**Ophthalmologic**	8 (31%)	Mild	3	Floater; short-sighted
		Moderate	3	Tearing/itchiness/redness
		Severe	2	Sensitivity to light; vision loss

**Gastrointestinal**	7 (27%)	Mild	1	Pain in the stomach
		Moderate	3	Cramping; heartburns; bloating
		Severe	3	Gastric ulcers

**Orthopedic**	7 (27%)	Mild	0	
		Moderate	4	Joint problems
		Severe	3	Amputation; prothesis

**Dental**	7 (27%)	Mild	1	Mouth sores
		Moderate	3	Loose teeth; sensitive gums; tooth
		Severe	3	decay Swollen gums; tooth pain

**Shorter stature than siblings**	5 (19%)			

**Other problems**	*3* (12%)	Mild	1	Dizziness
		Moderate	1	Dizziness
		Severe	1	Skin disease on the face

**Ear Nose Throat**	3 (12%)	Mild	1	Hearing loss
		Moderate	2	Nose bleeding
		Severe	0	

**Neurological**	3 (12%)	Mild	1	Numb feeling in limbs
		Moderate	2	Paralysis
		Severe	0	

**Psychological**	3	Mild	2	Fear of relapse
		Moderate	1	Anxiety
		Severe	0	

**Cognitive**	2 (8%)	Mild	2	Concentration/memory problems
		Moderate	0	
		Severe	0	

**Cardiac**	1 (4%)	Mild	0	
		Moderate	1	Awareness of heartbeat
		Severe	0	

**Renal**	1 (4%)	Mild	0	
		Moderate	1	Urinary tract infections
		Severe	0	

**Respiratory**	1 (4%)	Mild	1	Chest pain when it is cold
		Moderate	0	
		Severe	0	

**Endocrine**	0 (0%)			

**Subsequent malignancy**	0 (0%)			

### Puberty and Fertility

Median reported age at puberty (onset of pubic and axillary hair growth) was 14 years for females, and median reported age at puberty was 15 years for males. All ten female survivors menstruated, and in 90% of them, menses were regular. One woman was taking contraceptive drugs. Two female survivors and one male survivor were parents of a child. It had taken them less than two years to get pregnant (for one mother, the time to pregnancy was unknown). Nobody who had tried to conceive had experienced fertility issues.

### Performance Status

Table 3 shows the performance status of childhood cancer survivors. Forty-two percent of survivors were limited in their performance of personal care, physical work, social activities, daily chores, and school in the last four weeks. The number of restricted activities per survivor ranged from zero to four. The maximum grade of restricted activities per survivor was: never (58%), sometimes (23%), often (12%), and always (8%). Survivors were most limited in performing physical work (38%), personal care (23%), social activities (23%). Survivors diagnosed above 12 years were significantly more likely to have performance limitations (OR 12.4; p=0.015 [CI 95 1.83 – 83.77]).

**Table 3:** Performance status of childhood cancer survivors (n=26)

	Performance restricted in last four weeks				
	Total (n=11; 42%)	Always	Often	Sometimes	Never
**Physical work**	10 (38%)	1 (4%)	1 (4%)	8 (31%)	16 (62%)

**Personal care**	6 (23%)	0 (0%)	1 (4%)	5 (19%)	20 (77%)

**Social activities**	6 (23%)	1 (4%)	1 (4%)	4 (15%)	20 (77%)

**Daily chores**	4 (15%)	1 (4%)	1 (4%)	2 (8%)	22 (85%)

**School** (n=18)*	3 (17%)	1 (4%)	0 (0%)	2 (11%)	15 (83%)

**Employment** (n=5)**	0 (0%)	0 (0%)	0 (0%)	0 (0%)	5 (100%)

* 18 survivors attended school at time of interview

** 5 survivors were employed at time of interview

### Information about Late Effects at MTRH

Only 31% of survivors recalled being informed about the possibility of future late effects of cancer treatment at MTRH. Among them, 75% could recall which topics had been discussed. Physical late effects (100%) were more often mentioned than psychosocial late effects (17%). Half of all survivors (50%) shared that they worried about the late effects of cancer treatment. Almost all survivors (89%) would have liked to receive more information about the late effects of cancer treatment at the hospital.

### Preferred Follow-Up

According to the medical records, 92% of survivors had been in follow-up care after completion of treatment, although 17% of them only within the first half year. All survivors thought attending follow-up clinic was important, and 91% thought it was necessary. After final discharge, survivors considered it best to plan a future follow-up visit: within half a year (27%), within a year (58%), between one and five years (8%), after five years (4%), or never (4%). Survivors would prefer to go for follow-up to MTRH (92%) or a regional hospital (8%). The nearest county hospital was informed about their previous cancer history according to 15% of survivors, while 54% of survivors stated that they would like the county hospital to know about it. Reasons mentioned by 23% of survivors why they did not disclose their cancer history to a regional hospital were a negative attitude of healthcare providers towards childhood cancer, the reluctance of the survivor to recall past experiences, the lack of knowledge and capacity to take care of childhood cancer in regional facilities, and the wish to keep childhood cancer care centralized in one hospital. Fifteen percent of survivors avoided visiting hospitals because of their adverse experiences during treatment.

## Recommendations for Guidance of Survivors

Survivors (92%) shared various recommendations on follow-up care, addressing medical, community, psychosocial, and social reintegration aspects (Table 4).

**Table 4:** Topics highlighted in survivor recommendations about guidance and follow-up after treatment

	Mode of delivery	Content
**Hospital-provided survivorship care**	Clinic visits	Educate and engage parents about aftercare
	Home visits	Provide hope, spiritual and psychological support for survivors
	Phone calls	
	Peer discussion meetings	

**Childhood cancer awareness strategies**	Community awareness	Address that cancer is not a fatal disease

**Social reintegration improving practices**	Home visits	Counsel on coping with community life
	Encourage a good caregiver-survivor relationship	
	Provide job opportunities	

## Discussion

We present an overview of self-reported late effects among twenty-six Kenyan childhood cancer survivors. Most survivors were managed for a hematological malignancy and were treated with chemotherapy only. Seventy-three percent of childhood cancer survivors reported late effects: most prevalent were pain, fatigue, eye, orthopedic, gastrointestinal and dental problems. No subsequent malignancy or endocrine disturbances were reported. Few survivors were coping with psychological or cognitive issues. Forty-two percent of survivors were limited in their daily life performance. Survivors restricted in daily life experienced more self-reported late effects, and performance limitations were reported more often in patients diagnosed at an older age. Most participants felt they lacked knowledge about survivorship issues. They were motivated to attend follow-up frequently, preferably at MTRH, and expressed the wish to interact with their peers in group discussions.

The prevalence of self-reported late effects in our study population corresponds with findings from other studies based on self-reported outcomes.^33^-^35^ This type of cross-sectional study provides a rough estimate of long-term morbidity in the absence of complex infrastructure to undertake robust cohort studies. Such large cohorts have been established in the USA, Brazil, India, and the Netherlands, all on different continents, but not yet in Africa.^10^,^29^,^30^,^36^ Prevalence of self-reported late effects varied between 62% in Norway, 79% in Australia and 85% in the United Kingdom.^33^-^35^ Fatigue has repeatedly emerged as a major contributor to late effect burden, as seen in this Kenyan sample.^33^-^35^ It is also a known risk factor for the experience of pain, the most reported symptom in this study.^37^ A systematic review on chronic cancer-related pain found pain in five to 75% of survivors.^37^ In general, few studies have assessed pain with a validated instrument, hampering the interpretation of the wide variation between reported prevalences.^38^ We explored pain by asking respondents for its presence, nature (recurrent versus longstanding), and severity (mild, moderate, or severe). In many of the Kenyan survivors, pain resulted from back aches. Other orthopedic symptoms, such as joint pains, were reported in 27% of survivors. Underlying causes were not explored, but musculoskeletal issues may be affected by low bone mineral density, commonly occurring in survivors, especially after ALL treatment.^16^,^39^ African childhood cancer patients could be at risk of this particular long-term morbidity, due to malnourishment affecting the bone density in the general population before treatment.^16^,^39^ In contrast with other self-reported survivor studies, few cognitive or psychological problems were found.^33^-^35^,^40^ There were also no endocrine disorders reported, which could be attributed to the absence of brain tumor survivors in our study sample.^35^ It should be considered that mental health stigma in Kenya may hinder self-report of these conditions.^41^ Additionally, due to limited awareness among survivors of potential psychosocial effects and its less noticeable nature combined with a lack of protocolized screening, healthcare providers often fail to recognize signs of psychosocial late effects.^42^

Although the provided low-risk treatment in Kenya results from practicality rather than a deliberate choice, the relative lack of seriously disabling morbidity suggests a beneficial consequence of the limited available resources. The absence of subsequent malignancies and endocrine disorders in our study may be related to limited radiotherapy usage as well as a short follow-up time.^43^,^44^ Nevertheless, the impact of self-reported late effects was illustrated by 42% of survivors who were at least sometimes limited in their performance at personal care, physical work, social activities, daily chores, or school. Therefore, providing tools and strategies to support Kenyan childhood survivors to reintegrate into society should be prioritized.

Only 35% of survivors attended the follow-up clinic within the last year prior to the interview. Well-informed survivors and survivors diagnosed at younger ages remained in care for longer. Trustworthy information about health risks after surviving childhood cancer provided by survivorship experts may enhance follow-up adherence.^45^ Digital storage of survivorship care plans have eased the exchange of personalized information and allowed healthcare providers to keep track of the follow-up schedule.^46^ Health insurance has previously been identified as a prerequisite for follow-up adherence, in the HIC and LMIC settings.^47^,^48^ The potential benefit duration from parental health insurance is longer for young children than adolescents. It may contribute to the difference in follow-up time between the two in our study. Klosky et al. suggested that patients living closer to the hospital were likelier to quit follow-up since they could access care on demand. In contrast, those living far away prioritized their checkups because of the required preparations to attend the clinic according to schedule. Indeed, most survivors in this study were health-insurance subscribers but counter-intuitively lived far away and within many hours of traveling from MTRH. Nevertheless, despite the limited information they had received about the subject, they unanimously had a positive attitude towards the importance of follow-up.

Our study was limited by a small sample size due to the lack of valid contact details of many survivors. Phone numbers available often belonged to survivors whose condition required medical attention and who were intrinsically motivated to attend follow-up care or participate in peer support activities. The reported conditions are subjectively identified, thus were not confirmed by physical examination or additional investigations, and were not compared to a control group. As a result, the self-reported prevalence of late effects in this cohort may not equate to the actual prevalence. Future research on a larger population of childhood cancer survivors from LMIC is needed to have sufficient statistical power to identify risk groups. Assessment of relevant outcomes with internationally acknowledged measurement instruments will be fundamental to validate our results.^49^

Kenyan childhood cancer survivors generally reported similar late effects in frequency, nature and severity as other survivors worldwide. Pain appeared to be a profound symptom, while psychological and cognitive morbidity was less apparent. With survival improving and treatment options intensifying, Kenya will soon be confronted by an increasing number of survivors affected by late effects. Context-sensitive guidelines could assist healthcare providers in identifying, monitoring and treating late effects. Survivors should be involved in developing tailored follow-up infrastructure that considers the logistic and financial challenges of the LMIC setting. Healthcare facilities need to keep records of childhood cancer patients after treatment completion and target those who discontinue care. This will enable healthcare providers to reach survivors with relevant information about survivorship and to facilitate access to available services to manage late effects.
